# Robot-Assisted Anderson–Hynes Pyeloplasty for Lower-Moiety Ureteropelvic Junction Obstruction in an Incomplete Duplex Collecting System Presenting as Dietl’s Crisis: A Case Report

**DOI:** 10.3390/children13070934

**Published:** 2026-07-16

**Authors:** Dimitrios Deligiannis, Panagiotis Mitsos, Anna Papakonstantinou, Spyridon Skoufias, Aris Kaltsas

**Affiliations:** 1 Third Department of Urology, Attikon University Hospital, School of Medicine, National and Kapodistrian University of Athens, 12462 Athens, Greece; ddelijohn@med.uoa.gr; 2 Department of Urology, General Children’s Hospital “Agia Sofia”, Thivon 1 and Papadiamantopoulou, 11527 Athens, Greece; mitsospanagiotis@yahoo.com (P.M.); annapapak86@gmail.com (A.P.); 3 First Urology Clinic, IASO General Clinic, IASO Hospital, 15123 Athens, Greece; spyskouf@hotmail.com

**Keywords:** Dietl’s crisis, lower-moiety ureteropelvic junction obstruction, incomplete duplex collecting system, bifid ureter, crossing vessels, robot-assisted pyeloplasty, MAG3 renography

## Abstract

**Highlights:**

**What are the main findings?**

- A normal interval ultrasound does not exclude intermittent (Dietl’s crisis) ureteropelvic junction obstruction.
- CT urography localized lower-moiety obstruction within an incomplete duplex system and identified crossing vessels.

**What are the implications of the main findings?**

- MAG3 confirmed functionally significant obstruction with preserved overall renal function.
- Robot-assisted Anderson–Hynes pyeloplasty was associated with no symptom recurrence and improved drainage at early 3-month follow-up.

**Abstract:**

Background/Objectives: Intermittent ureteropelvic junction obstruction (UPJO), classically termed Dietl’s crisis, may be missed when imaging is obtained outside symptomatic periods. The challenge is amplified in duplicated collecting systems, where the obstructed moiety may not be recognized on screening ultrasonography. Case Presentation: A 14-year-old boy presented with recurrent severe right flank pain triggered by heavy fluid intake, associated with nausea and vomiting and separated by symptom-free intervals. An initial renal ultrasound performed between attacks was normal. One week later, the same pain pattern recurred together with a febrile urinary tract infection. Computed tomography urography (CTU) demonstrated an incomplete right duplex collecting system with a bifid ureter, marked hydronephrosis of the lower moiety, delayed contrast excretion, and focal narrowing at the lower-moiety ureteropelvic junction adjacent to one crossing artery and one crossing vein. Diuretic ^99m^Tc-mercaptoacetyltriglycine (MAG3) renography documented obstructive drainage, with a post-furosemide drainage half-time (T1/2) > 20 min, differential renal function of 51% on the right, and split moiety function of 31% (upper) and 20% (lower). The patient underwent transperitoneal robot-assisted dismembered Anderson–Hynes pyeloplasty of the lower moiety with the reconstructed ureteropelvic junction repositioned anterior to the crossing vessels; a 6 Fr × 26 cm double-J stent was placed and subsequently removed at 4 weeks postoperatively. Total skin-to-skin operative time was 75 min, estimated blood loss < 100 mL, and the postoperative course was uneventful. At early 3-month follow-up, the patient remained free of Dietl-type episodes, and ultrasonography showed marked reduction in lower-moiety hydronephrosis. Selective postoperative CTU, obtained because of the unusual bifid anatomy, demonstrated patent drainage. Conclusions: A normal interval ultrasound should not exclude intermittent UPJO when the history is stereotypical, and cross-sectional plus functional imaging is decisive when duplex anatomy and crossing vessels coexist.

## 1. Introduction

Dietl’s crisis refers to episodic abdominal or flank pain caused by intermittent ureteropelvic junction obstruction (UPJO), often precipitated by increased diuresis. In pediatric practice, this presentation remains under-recognized and is frequently associated with diagnostic delay despite preserved renal parenchyma and salvageable renal function at the time of diagnosis [1,2,3,4].

A central diagnostic pitfall is the transient nature of the obstruction. Hydronephrosis may be pronounced during painful episodes and minimal or absent on interval ultrasonography, creating false reassurance and delaying referral. Case-based imaging reports and pediatric series have shown that ultrasonography and even functional studies may appear equivocal between attacks, whereas imaging obtained during or soon after symptoms can be far more informative [2,3,5].

Diuretic renography remains fundamental for functional evaluation; however, intermittent obstruction may produce variable renographic patterns. Sparks et al. emphasized that cortical retention, drainage curve morphology, and differential renal function should be interpreted together rather than relying on the drainage half-time (T1/2) alone, particularly in Dietl’s crisis [5]. Current pediatric guidance similarly recommends standardized hydration and appropriate bladder management to improve interpretability of ^99m^Tc-mercaptoacetyltriglycine (MAG3) studies [6,7].

UPJO occurring within a duplicated collecting system is uncommon and most often affects the lower moiety [8,9,10,11,12]. Duplex anatomy can obscure the site of obstruction, particularly when duplication is incomplete and the ureteral confluence is close to the affected ureteropelvic junction. In such cases, detailed preoperative anatomical definition is essential because reconstructive strategy depends on available ureteral length and the relationship of the lower-moiety pelvis to the non-obstructed moiety [9,10,11,12].

Crossing lower-pole vessels are another clinically important variable. They are a well-recognized contributor to symptomatic UPJO, especially in older children and adolescents, and may help explain intermittent symptoms during periods of increased urinary flow [13,14,15]. Minimally invasive reconstruction, including robot-assisted pyeloplasty, is increasingly used in pediatric and adolescent UPJO and offers precise dissection and suturing in anatomically complex cases [12,16,17,18,19].

This case report describes a 14-year-old boy with intermittent Dietl-type symptoms caused by lower-moiety UPJO in an incomplete duplex collecting system with crossing vessels, managed by robot-assisted dismembered Anderson–Hynes pyeloplasty. This case is reported in accordance with the CARE (Case Report) reporting guideline; the completed CARE checklist is provided in the Supplementary Materials (File S1).

## 2. Case Presentation

### 2.1. Patient Information and Symptom Pattern

A 14-year-old boy was referred for evaluation of recurrent right-sided flank pain. The episodes had become progressively more frequent over the preceding 1–2 months. The attacks were stereotyped: severe right flank pain occurred shortly after heavy fluid intake, was associated with nausea and vomiting, and resolved spontaneously. Between episodes he was symptom-free.

No prenatal hydronephrosis, chronic medication use, allergies, previous urological surgery, or relevant family history was identified.

### 2.2. Acute Episodes and Intercurrent Febrile UTI

An initial renal ultrasound obtained outside an acute painful episode demonstrated no hydronephrosis. During one severe episode, the patient presented with right flank pain, nausea, and vomiting. Physical examination showed no right costovertebral-angle tenderness. Routine laboratory testing was within normal limits, and urinalysis was negative. He was discharged with medical therapy.

Approximately 1 week later, the patient re-presented with recurrent right flank pain, fever to 39 °C, and chills. Laboratory testing demonstrated leukocytosis, and urinalysis revealed pyuria and bacteriuria. He was diagnosed with a febrile urinary tract infection (UTI) consistent with acute pyelonephritis and started on empiric intravenous cefuroxime and gentamicin. Urine culture yielded Escherichia coli that was susceptible to all tested agents, and therapy was subsequently streamlined accordingly. Blood cultures remained sterile. He completed a 10-day antibiotic course in total, transitioning from intravenous therapy to oral cefuroxime once clinically improved and able to tolerate enteral treatment.

### 2.3. Cross-Sectional Imaging (CT Urography)

To better define the collecting-system anatomy and identify the site of obstruction at the moiety level, contrast-enhanced CT urography (CTU) was obtained. A formal hydration- or diuresis-provoked ultrasound was not performed. Because the patient had recurrent stereotyped diuresis-triggered pain, a symptomatic ultrasound had already demonstrated SFU grade 3 hydronephrosis confined to the lower collecting system, and the anatomy was suspected to be complex, CTU was selected to define the moiety-specific obstruction, ureteral confluence, delayed excretion, and vascular relationships in a single examination. Functional MR urography was considered as a radiation-sparing alternative. In this patient, CTU was chosen because it was immediately available, provided high-spatial-resolution definition of the incomplete duplex anatomy, demonstrated delayed lower-moiety excretion, and delineated the crossing artery and vein for operative planning. The use of CTU was therefore individualized and justified by the need for rapid moiety-specific anatomical and vascular mapping. In this report, incomplete duplication refers to a Y-shaped bifid ureter draining through a single distal ureteral orifice. CTU demonstrated an incomplete right duplex collecting system with a bifid ureter. The upper moiety was nondilated and drained normally. The lower moiety showed marked pelvicalyceal dilatation, delayed contrast excretion, and focal narrowing at the lower-moiety ureteropelvic junction (UPJ). On CTU, the maximal lower-moiety pelvic diameter was approximately 35 mm. One crossing lower-pole artery and one crossing lower-pole vein were identified adjacent to the narrowed segment, supporting a likely extrinsic component to the intermittent obstruction. Targeted Doppler ultrasound to assess the crossing vessels was not performed; the vascular anatomy was instead defined by contrast-enhanced CTU and confirmed intraoperatively. No urolithiasis was identified on definitive cross-sectional imaging.

A preceding ultrasound performed during recurrent symptoms demonstrated Society for Fetal Urology (SFU) grade 3 hydronephrosis confined to the lower collecting system. The anteroposterior diameter of the lower-moiety renal pelvis was 29 mm on symptomatic ultrasonography. SFU grading was therefore attributed to ultrasonography, whereas CTU was used to define moiety-specific anatomy, delayed excretion, the level of narrowing, and the relationship of the lower-moiety UPJ to the crossing vessels.

**Figure 1:**
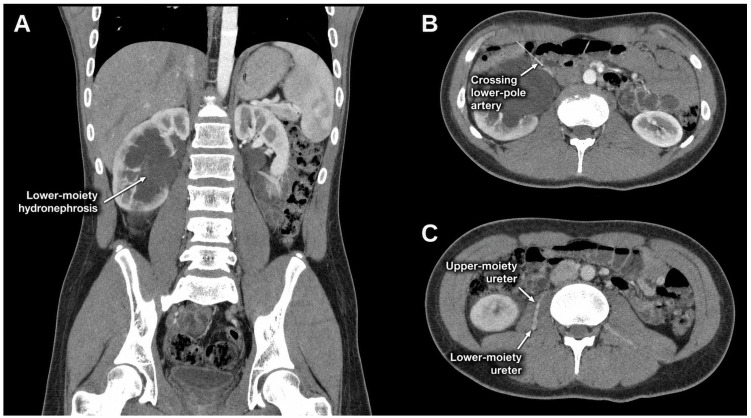
Preoperative CT urography of the right urinary tract. Representative CTU images demonstrated: (**A**) coronal delayed urographic-phase image showing hydronephrosis confined to the lower moiety with delayed excretion; (**B**) axial nephrographic-phase image showing a crossing lower-pole renal artery at the level of the lower-moiety ureteropelvic junction; the accompanying lower-pole vein was identified on the CTU series and confirmed intraoperatively; and (**C**) axial image demonstrating incompletely duplicated/bifid ureters. CTU localized the obstruction to the lower-moiety UPJ and showed no urolithiasis.

CTU included nephrographic and delayed excretory-phase acquisitions, allowing assessment of parenchymal enhancement, collecting-system opacification, drainage, and vascular relationships. The preoperative CTU findings are shown in Figure 1.

### 2.4. Functional Assessment and Diagnostic Synthesis

Diuretic renography with ^99m^Tc-MAG3 demonstrated impaired drainage consistent with obstruction. The study was interpreted in conjunction with the clinical history and CTU anatomy, following standard pediatric diuretic-renography principles [6,7]. Attempts were made to retrieve the complete acquisition protocol; however, the archived report did not specify the hydration protocol, furosemide timing, or bladder-drainage method. For this reason, the reported T1/2 > 20 min was not interpreted as a standalone diagnostic criterion. The functional diagnosis was based on a multimodal framework: stereotyped diuresis-triggered symptoms, CTU localization of lower-moiety UPJ narrowing with delayed excretion, renographic drainage-curve morphology with persistent post-diuretic retention, and preserved but potentially at-risk renal function.

The post-furosemide drainage curve did not reach 50% clearance during the post-diuretic acquisition; therefore, the half-time was reported qualitatively as T1/2 > 20 min rather than as a precise numeric value. Overall differential renal function was 51% for the right kidney and 49% for the left kidney. Segmental right renal function was estimated as 31% for the upper moiety and 20% for the lower moiety.

**Figure 2:**
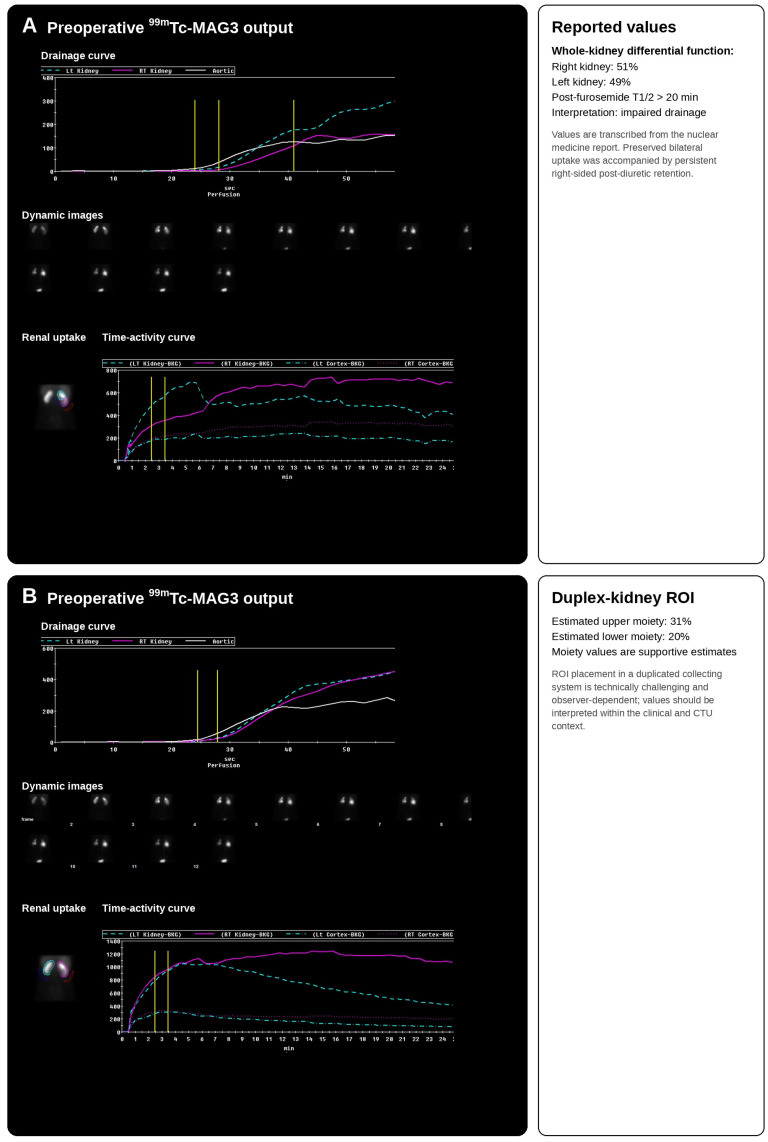
Preoperative ^99m^Tc-MAG3 diuretic renography. (**A**) Whole-kidney dynamic images, renal uptake image, and renographic curves showing preserved bilateral uptake with impaired right-sided post-diuretic drainage. Whole-kidney differential renal function was 51% for the right kidney and 49% for the left kidney. The post-furosemide drainage curve did not reach 50% clearance during the acquisition window; therefore, T1/2 was reported as >20 min. (**B**) Right duplex-kidney region-of-interest analysis used to estimate segmental contribution of the upper and lower moieties. Estimated right-sided segmental function was 31% for the upper moiety and 20% for the lower moiety. Moiety-specific values were considered supportive ROI-dependent estimates in the setting of duplex anatomy. Abbreviations: MAG3, mercaptoacetyltriglycine; ROI, region of interest; T1/2, half-time.

Moiety-specific split function was estimated by drawing separate regions of interest (ROIs) over the upper and lower right renal moieties on summed functional images, guided by the CTU-defined anatomy and renographic morphology, with background correction according to the nuclear medicine software. Moiety-specific ROI segmentation was performed by a single experienced nuclear medicine physician, using CTU-defined anatomy to guide separation of the upper and lower moieties; no formal inter-observer agreement analysis was performed, and no standardized, validated protocol for moiety-specific ROI placement was prospectively applied. This approach was nonetheless consistent with the principle that lower-moiety drainage in duplex systems should be interpreted with careful moiety-level ROI analysis, as emphasized by Kim et al. [20]. Because ROI placement in a duplicated collecting system is technically challenging and observer dependent, the upper-/lower-moiety values were interpreted as supportive estimates rather than as the sole indication for surgery. The time to peak activity (Tmax) was 6 min for the right kidney and 4.5 min for the left kidney; this parameter was not interpreted in isolation, because an early parenchymal peak may remain relatively preserved despite downstream collecting-system obstruction. The diagnosis was based on the combined findings of persistent post-diuretic retention, obstructive drainage-curve morphology, preserved but at-risk function, CTU localization of lower-moiety UPJ narrowing, and the stereotyped Dietl-type symptom pattern. The preoperative renographic images and drainage curves are shown in Figure 2.

Taken together, the clinical history and multimodal imaging supported the diagnosis of right lower-moiety UPJO in an incomplete duplex collecting system, presenting as Dietl’s crisis and associated with crossing lower-pole vessels. The diagnostic sequence was instructive: the initial normal interval ultrasound was misleading, CTU defined the duplicated anatomy and vascular relationships, and MAG3 established functionally significant obstruction despite preserved overall renal function. A summary of the clinical trajectory, diagnostic evaluation, and management is provided in Table 1.

### 2.5. Operative Management

The indication for surgery was based on the combination of recurrent stereotyped diuresis-triggered flank pain, a documented febrile UTI, symptomatic SFU grade 3 lower-moiety hydronephrosis, CTU evidence of delayed lower-moiety excretion with focal UPJ narrowing adjacent to crossing vessels, and obstructive drainage-curve morphology on MAG3 with preserved overall renal function. The estimated lower-moiety split function was considered supportive but was not the sole indication for reconstruction.

A robot-assisted transperitoneal approach was selected because the patient was an adolescent with adequate working space, the anatomy was complex because of incomplete duplication and crossing vessels, and the operation required precise dissection, vessel preservation, spatulation, and intracorporeal suturing for a dependent tension-free anastomosis. Open and conventional laparoscopic repair remain valid alternatives; in this case, the robotic platform was used as an enabling reconstructive tool rather than because it is inherently superior in all paediatric UPJO cases.

A transperitoneal robot-assisted dismembered pyeloplasty based on Anderson–Hynes principles was performed using the da Vinci Xi system, four robotic ports, and one assistant port. After abdominal entry, the ascending colon was mobilized along the right Toldt line, and a Kocher maneuver was performed to expose the retroperitoneum and renal pelvis.

**Figure 3:**
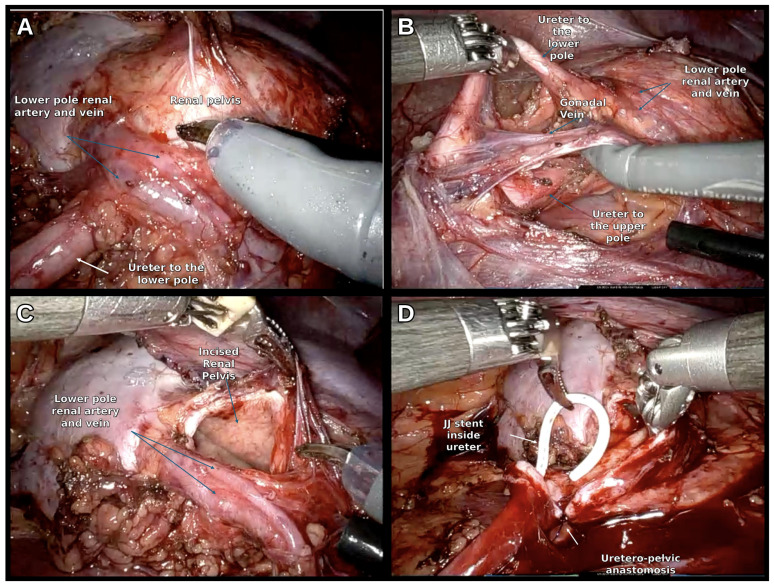
Intraoperative robotic lower-moiety Anderson–Hynes pyeloplasty. (**A**) Lower-moiety pelvis and ureter with a crossing lower-pole artery and vein at the lower-moiety ureteropelvic junction. (**B**) Bifid ureteral anatomy after dissection to the ureteral confluence, with separate upper- and lower-moiety ureters and the gonadal vein labeled. (**C**) Incised lower-moiety renal pelvis during dismembered reconstruction with preservation of the crossing vessels. (**D**) Completed ureteropelvic anastomosis after anterior transposition relative to the crossing vessels, with a double-J stent in situ.

The bifid ureter was identified and dissected caudally to the ureteral confluence and cranially toward the separate lower-moiety pelvis. Intraoperatively, one lower-pole artery and one lower-pole vein were seen crossing the lower-moiety ureteropelvic junction, concordant with the CTU findings. The stenotic lower-moiety UPJ was dismembered. A small amount of redundant pelvis was excised. The opened UPJ and proximal ureter were inspected intraoperatively, and no gross intraluminal lesion, fibroepithelial polyp, or stone was identified. The lower-moiety ureter was spatulated, and a dependent, tension-free ureteropelvic anastomosis was constructed with continuous 4-0 Vicryl suture. The reconstructed UPJ and ureter were transposed anterior to the crossing vessels while preserving them. Representative intraoperative anatomy and reconstruction are shown in Figure 3.

Although no formal intraoperative measurement of the UPJ-to-confluence distance was obtained, intraoperative review of the operative field suggested a distance of approximately 4–5 cm. This placed the anatomy closer to the group in which dismembered pyeloplasty is technically feasible, rather than the very short lower-moiety ureter described by VanderBrink et al. [9] and the ≤3 cm group in Liu et al. [11], in which pyeloureterostomy was commonly selected. Intraoperative confirmation of a dependent, tension-free ureteropelvic anastomosis ultimately guided the decision to proceed with Anderson–Hynes reconstruction rather than pyeloureterostomy.

A 6 Fr × 26 cm internal double-J stent was left across the anastomosis, and a drain was placed and removed after 24 h. The total skin-to-skin operative time was 75 min, including robotic docking. Estimated blood loss was <100 mL, and no intraoperative complications were reported.

### 2.6. Postoperative Course and Follow-Up

Recovery was uneventful, and the patient was discharged on postoperative day 2. The double-J stent was removed 4 weeks after surgery.

Maximum completed follow-up at the time of manuscript preparation was approximately 3 months. Because follow-up was limited to 3 months, postoperative outcomes were considered early. The primary early endpoints were absence of recurrent Dietl-type episodes and improvement/reduction in lower-moiety hydronephrosis on ultrasonography. During this early follow-up period, the patient had no recurrence of Dietl-type episodes. Ultrasonography demonstrated marked reduction in lower-moiety hydronephrosis. At 3 months, the lower-moiety pelvic AP diameter decreased from 29 mm preoperatively to 11–12 mm. The postoperative MAG3 study was obtained selectively because of the unusual duplex anatomy and prior moiety-specific obstruction; it was not intended as a routine early surveillance test. Given the limitations of early postoperative renography and possible residual collecting-system dilatation, it was interpreted only as supportive evidence of improved drainage compared with baseline. Repeat CTU demonstrated patent urinary drainage through the bifid system without recurrent hydronephrosis. The 3-month CTU was obtained selectively because of the unusual duplex anatomy, prior crossing-vessel compression, and the need to document postoperative moiety-specific drainage after reconstruction. Further follow-up is planned at 1 year with clinical assessment and renal ultrasonography. MAG3 renography or fMRU will be reserved for recurrent pain, febrile UTI, worsening hydronephrosis, or uncertainty regarding moiety-specific drainage.

## 3. Discussion

This case illustrates how intermittent obstruction, duplex anatomy, and crossing vessels can converge to delay diagnosis and complicate surgical planning. The clinical history was highly suggestive of Dietl’s crisis: abrupt flank pain triggered by increased diuresis, associated nausea and vomiting, and complete resolution between attacks. Alagiri and Polepalle emphasized that this presentation is frequently missed in children, often leading to prolonged diagnostic delay despite good outcomes after pyeloplasty [1]. Chen et al. similarly showed that attack characteristics and imaging severity during symptoms may correlate with functional impairment, underscoring the value of timely recognition [3].

The most important practical pitfall is over-reliance on interval ultrasound. Ultrasound remains the appropriate first-line study in children with suspected hydronephrosis, but in intermittent UPJO the collecting system may decompress between attacks. Lahoud et al. reported striking fluctuation in hydronephrosis during and after symptomatic episodes, a pattern that closely mirrors the diagnostic challenge in the present case [2]. For that reason, a normal interval ultrasound should not outweigh a convincing symptom history. When the clinical pattern is stereotyped, repeat imaging during symptoms or escalation to functional and cross-sectional evaluation is warranted [1,2,3,5].

Renography in Dietl’s crisis also requires careful interpretation. Sparks et al. demonstrated that intermittent UPJO may produce heterogeneous renographic patterns, including clearly obstructive scans, cortical retention, or occasionally nondiagnostic studies depending on the timing of imaging [5]. Contemporary pediatric guidance therefore recommends that MAG3 interpretation integrate multiple parameters, including drainage curves, differential function, hydration status, and bladder management [6,7]. In this patient, MAG3 showed a clearly obstructive pattern with preserved overall right-sided function, strengthening the rationale for intervention. This decision was consistent with contemporary EAU/ESPU paediatric urology guidance, which supports pyeloplasty for symptomatic UPJO, including recurrent flank pain or UTI, and recognizes poor post-furosemide drainage, increasing anteroposterior pelvic diameter, and high-grade SFU dilatation as additional indications for intervention [21]. Lower-moiety hydronephrosis in duplex systems behaves dynamically and may present with obstructive drainage during Dietl’s crisis, underscoring the value of diuresis renography in this setting [20].

Duplex anatomy introduces additional layers of complexity. Lower-moiety UPJO within duplicated collecting systems is rare, and operative planning depends heavily on whether duplication is complete or incomplete and on how close the ureteral confluence lies to the obstructed UPJ [8,9,10,11,12]. Gonzalez et al. described lower-pole pelvi-ureteric junction obstruction in duplex systems as an uncommon but distinct entity requiring individualized repair [8]. VanderBrink et al. and Avlan et al. highlighted a key technical point: when the lower-moiety ureter is short because the confluence is proximal, pyeloureterostomy may be preferable to standard dismembered pyeloplasty; when sufficient lower-moiety ureteral length is available, dismembered reconstruction remains feasible [9,10]. This distinction is clinically relevant because a short lower-moiety ureter between the UPJ and ureteral confluence may favor pyeloureterostomy rather than dismembered pyeloplasty. Liu et al. classified patients by UPJ-to-confluence distance, with pyeloureterostomy commonly used when the distance was ≤3 cm and laparoscopic pyeloplasty used when the distance was >3 cm [11]. In the present case, the confluence was sufficiently caudal to permit a dependent, tension-free lower-moiety Anderson–Hynes anastomosis. Recent series likewise confirm that duplex UPJO is uncommon and that the choice between dismembered pyeloplasty and pyeloureterostomy should be anatomy-driven, with comparable outcomes [22]; lower-moiety obstruction in a duplex system has also been reported with atypical presentations [23].

That framework helps explain why preoperative anatomical mapping was so important in the present case. CTU demonstrated an incomplete duplex system with a bifid pelvis and bifid ureter, clearly localized the obstruction to the lower moiety, and delineated the adjacent crossing vessels. Intraoperative findings then confirmed adequate lower-moiety ureteral length for tension-free Anderson–Hynes reconstruction, allowing excision of the narrowed segment and dependent re-anastomosis rather than pyeloureterostomy. Thus, duplex anatomy did not preclude dismembered pyeloplasty; instead, it required anatomy-specific confirmation that a dismembered repair was technically sound.

Crossing vessels were another central feature of this case and likely contributed to symptom intermittency. Crossing lower-pole vessels are a recognized cause of extrinsic UPJO, particularly in older symptomatic children and adolescents [13,14,15]. Gupta et al. observed that preoperative ultrasound and dynamic renal scans do not reliably predict the presence of crossing vessels, which are often diagnosed intraoperatively [13]. Parikh et al. showed that magnetic resonance urography (MRU) can identify crossing vessels with high agreement with surgical findings, while Wong et al. found useful diagnostic performance for both color Doppler ultrasound and MR urography when vascular anatomy is specifically interrogated [14,15]. In the present case, CTU directly depicted one crossing artery and one crossing vein adjacent to the narrowed lower-moiety UPJ, allowing the operative plan to incorporate vessel-preserving anterior transposition from the outset.

The differential diagnosis of recurrent flank pain in an adolescent is broad and includes nephrolithiasis, pyelonephritis, nonobstructive hydronephrosis, musculoskeletal pain, and gastrointestinal causes. In this patient, the febrile UTI was clinically important but did not fully explain the recurrent stereotyped attacks, because the Dietl-type pain pattern preceded the documented infection, was reproducibly triggered by heavy fluid intake, and resolved between episodes. Definitive CTU localized delayed excretion and focal narrowing to the lower-moiety UPJ and showed no urolithiasis. The overall pattern therefore supported intermittent lower-moiety UPJO rather than infection or stone disease as the primary explanation for the recurrent attacks. Intermittent UPJO may also be mimicked by other lesions and is sometimes unmasked only by provocative, hydration-augmented imaging, reinforcing the need for a high index of suspicion [24]. Likewise, rare intraluminal lesions such as fibroepithelial polyps can cause pediatric UPJO and may be identified only at operation [25].

The literature supports individualized minimally invasive reconstruction for lower-moiety UPJO in duplicated systems. Belmont et al., in a multi-institutional pediatric minimally invasive series, tailored repair to the anatomic variant and reported an overall success rate of 93% with low complication rates [12]. Case reports and smaller series have also demonstrated the feasibility of robotic pyeloureterostomy or pyeloplasty when matched appropriately to anatomy [11,26]. The present case aligns with that experience: robotic reconstruction was selected because it allowed precise dissection around the duplex ureter and crossing vessels and facilitated a meticulous intracorporeal anastomosis.

Robotic pyeloplasty in pediatric and adolescent patients is supported by a growing observational evidence base, although the evidence remains heterogeneous. In a systematic review and meta-analysis, Greenwald et al. reported a mean success rate of 95.4% and a mean overall complication rate of 12% for pediatric robot-assisted pyeloplasty [16]. Esposito et al. likewise concluded that robotic pyeloplasty is safe and effective in children, including more complex cases, while noting the limitations of retrospective study design and variable follow-up [17]. More recent multi-institutional comparisons continue to demonstrate high success and low morbidity for robot-assisted pyeloplasty, including in the youngest patients [27]. Minnillo et al. reported durable long-term results in children and young adults [18], and comparative multicenter data suggest broadly similar functional outcomes among open, laparoscopic, and robotic approaches, with differences more evident in operative time, hospital stay, and resource use than in ultimate success [19]. This is consistent with current EAU paediatric urology guidance, which reports comparable success and complication rates for open and minimally invasive (including robot-assisted) pyeloplasty [21]. The robotic platform therefore should be viewed as a valuable reconstructive tool rather than an intrinsically superior option in every case.

Imaging selection in adolescents with suspected intermittent UPJO should balance diagnostic yield against radiation exposure. CTU was decisive here because it simultaneously defined the duplicated anatomy, localized the obstructed moiety, demonstrated delayed excretion, and showed the crossing vessels. Nevertheless, CT carries ionizing radiation. Bombinski et al. demonstrated that low-dose CT urography can preserve diagnostic image quality in children with congenital urinary tract abnormalities [28], and Ferrero et al. emphasized the principles of justification and dose optimization in nephrourologic CT imaging [29]. Functional MR urography (fMRU) offers a radiation-sparing alternative; Damasio et al. and Al-Shaqsi et al. reported good agreement between MR urography and scintigraphy for split renal function in pediatric congenital urinary tract anomalies and UPJO cohorts [30,31]. Where available, MRU or fMRU may be particularly attractive in adolescents, but CTU remains justified when complex anatomy and vascular relationships must be defined quickly and clearly.

The postoperative repeat CTU should be interpreted in this context. Although routine CT-based surveillance is not desirable in adolescents because of cumulative radiation exposure, this examination was used selectively to confirm unobstructed drainage through a reconstructed lower-moiety UPJ in an anatomically complex bifid system with crossing vessels. Future follow-up should prioritize ultrasonography and functional assessment, and functional MR urography may be considered when cross-sectional anatomical and functional reassessment is required without additional ionizing radiation [28,29,30,31].

The incremental educational value of this case lies in the convergence of several uncommon features: intermittent Dietl-type symptoms, lower-moiety UPJO in an incomplete duplex system, dual crossing vessels, initially normal interval ultrasonography, moiety-level functional assessment, and anatomy-driven selection of dismembered pyeloplasty rather than pyeloureterostomy.

Overall, the present case reinforces a practical clinical message: a single normal ultrasound should not dismiss a convincing history of recurrent diuresis-triggered flank pain. Once duplex anatomy is suspected, identifying the symptomatic moiety, the level of ureteral confluence, and the presence or absence of crossing vessels becomes essential for selecting the correct reconstructive strategy. Table 2 summarizes selected reports of lower-moiety UPJO in duplicated collecting systems managed by minimally invasive repair, situating the present case within this limited but growing body of literature.

Limitations. This report has several limitations. First, it describes a single patient, and the diagnostic and operative strategy cannot be generalized to all patients with Dietl’s crisis or duplex collecting-system anatomy. Second, completed follow-up is limited to approximately 3 months; the absence of recurrent symptoms and improved imaging are early findings, and longer surveillance is required to confirm durability. Third, the archived MAG3 report did not include all acquisition details, including hydration protocol, furosemide timing, and bladder-drainage method; therefore, T1/2 was not interpreted in isolation. Fourth, moiety-specific split function in a duplicated system is ROI-dependent, and single-observer analysis without formal interobserver validation limits the precision of the 31% and 20% estimates. Fifth, no formal intraoperative measurement of the UPJ-to-confluence distance was obtained, although the anatomy permitted a tension-free anastomosis. Future cases should prospectively document this distance because it directly informs the choice between dismembered pyeloplasty and pyeloureterostomy.

## 4. Conclusions

Dietl’s crisis should remain in the differential diagnosis for adolescents with stereotyped recurrent flank pain, especially when attacks are triggered by increased fluid intake and accompanied by nausea or vomiting. A normal ultrasound obtained between episodes does not exclude clinically important intermittent UPJO.

In the setting of an incomplete duplex collecting system, CTU can clarify moiety-specific anatomy, localize the obstructed segment, and identify contributory crossing vessels, while diuretic renography confirms the functional significance of impaired drainage. When lower-moiety ureteral length is adequate, robot-assisted Anderson–Hynes pyeloplasty is a feasible anatomy-driven option. In this patient, robot-assisted Anderson–Hynes pyeloplasty was associated with no recurrence of Dietl-type episodes and improved drainage at early 3-month follow-up. Longer follow-up is required to confirm durability. A 1-year clinical and ultrasonographic follow-up visit is planned, with MAG3 renography or fMRU reserved for recurrent symptoms, febrile UTI, worsening hydronephrosis, or uncertainty regarding moiety-specific drainage.

## Figures and Tables

**Table 1:** Timeline of clinical course, diagnostic work-up, and management.

Timepoint	Clinical Events and Findings
~1–2 months before surgery	Recurrent self-limited right flank pain episodes; attacks increased in frequency; often precipitated by heavy fluid intake.
Initial interval evaluation	Renal ultrasound reportedly normal when obtained between attacks.
Severe symptomatic episode	Right flank pain with nausea/vomiting; no right costovertebral-angle tenderness; routine laboratory testing reportedly normal; urinalysis negative; discharged with medical therapy.
~1 week later	Febrile UTI with fever to 39 degrees C, chills, and flank pain; leukocytosis; pyuria and bacteriuria; treated with intravenous cefuroxime and gentamicin; urine culture positive; antibiotics adjusted.
~4 weeks later	Recurrent flank pain; ultrasound showed SFU grade 3 hydronephrosis confined to the lower collecting system.
Subsequent CTU	Incomplete duplication (bifid pelvis and bifid ureter); normal upper moiety; marked lower-moiety hydronephrosis with delayed excretion; focal lower-moiety UPJ narrowing adjacent to crossing artery and vein; no urolithiasis identified.
Subsequent MAG3	Obstructive drainage (T1/2 > 20 min); overall right differential renal function 51%; upper-moiety split function 31%; lower-moiety split function 20%; Tmax right 6 min, left 4.5 min.
Surgery	Transperitoneal robot-assisted dismembered Anderson–Hynes pyeloplasty; anterior transposition relative to crossing vessels; 6 Fr × 26 cm internal double-J stent; drain removed after 24 h; operative time 75 min; estimated blood loss < 100 mL.
Postoperative course	Uneventful recovery; discharge on postoperative day 2.
4 weeks postoperatively	Stent removal.
Early follow-up	Ultrasound: marked reduction in lower-moiety hydronephrosis; symptom-free.
~3 months postoperatively	No recurrent Dietl-type episodes; ultrasound showed marked reduction in lower-moiety hydronephrosis. Selective CTU showed patent drainage through the bifid system. Selective MAG3, interpreted cautiously, suggested improved drainage compared with baseline. Lower-moiety renal pelvic AP diameter: 29 mm preoperatively → 11–12 mm at 3 months.
Planned 1-year follow-up	Clinical review and renal ultrasonography; MAG3 renography or fMRU only if recurrent Dietl-type pain, febrile UTI, worsening hydronephrosis, or uncertainty regarding moiety-specific drainage.

**Table 2:** Selected literature on lower-moiety UPJO in duplicated collecting systems and minimally invasive repair.

Author/Year	Study Type/Population	Duplication Type	Affected Moiety	Reconstruction	Follow-Up	Main Outcome
Gonzalez et al. (2006) [8]	Retrospective pediatric series; 5 lower-pole UPJO cases within duplex systems.	Complete and incomplete duplication	Lower moiety	Primarily dismembered pyeloplasty; 1 ureteric calycostomy.	Scintigraphic follow-up; duration not reported.	Improved drainage on follow-up radioisotope scans; no functional obstruction reported.
VanderBrink et al. (2009) [9]	Retrospective series; 8 patients.	Incomplete/Y-shaped bifid ureter	Lower moiety	Dismembered pyeloplasty when ureteral length was adequate; lower-pole-to-upper-pole pyeloureterostomy when the ureter was short.	1 year	Anatomy-driven procedure selection; no complications or obstruction of either moiety.
Avlan et al. (2010) [10]	Retrospective series; 7 patients.	Incomplete/Y-shaped bifid ureter	Lower moiety	Pyeloureterostomy in most patients; dismembered pyeloplasty in selected anatomy.	14 months	No complications; individualized reconstruction emphasized.
Liu et al. (2016) [11]	Retrospective pediatric series; 7 patients.	Incomplete/Y-shaped bifid ureter	Lower moiety	End-to-side pyeloureterostomy or laparoscopic pyeloplasty according to UPJ-to-confluence distance.	Not reported.	Hydronephrosis improved; reconstructive choice guided by confluence level and available ureteral length.
Belmont et al. (2021) [12]	Multi-institutional pediatric minimally invasive series; 41 patients.	Duplication anomaly; type variable/not specified	Lower moiety	Laparoscopic and robotic dismembered pyeloplasty, ureteropyelostomy, and combined procedures tailored to anatomy.	Not reported.	Overall surgical success 93%; low complication burden; minimally invasive repair feasible and safe when individualized.
Lima et al. (2017) [26]	Single pediatric case report.	Incomplete/Y-shaped bifid ureter	Lower moiety	Robot-assisted pyeloureterostomy.	Not reported.	Demonstrated technical feasibility of robotic reconstruction with postoperative sonographic improvement.

## Data Availability

The data supporting the findings of this study are contained within the article. Owing to patient privacy and confidentiality, the underlying clinical records are not publicly available but are available from the corresponding author upon reasonable request.

## Supplementary Materials

The following supporting information can be downloaded at: https://www.mdpi.com/article/10.3390/children13070934/s1, File S1: CARE Checklist (2013)—Completed. Reference [32] is cited in the Supplementary Materials.
