# Supplementary material for: Robot-Assisted Anderson–Hynes Pyeloplasty for Lower-Moiety Ureteropelvic Junction Obstruction in an Incomplete Duplex Collecting System Presenting as Dietl’s Crisis: A Case Report
Source: Children (Basel). 2026 Jul 16;13(7):934. doi: 10.3390/children13070934 (PMC13406795; doi:10.3390/children13070934)
Supplement: Supplementary file 1 [file children-13-00934-s001.zip › children-4389511-supplementary.pdf]

CARE Checklist (2013) — Completed

Manuscript: *Robot-Assisted Anderson–Hynes Pyeloplasty for Lower-Moiety Ureteropelvic Junction Obstruction in an Incomplete Duplex Collecting System Presenting as Dietl's Crisis: A Case Report*

Reporting checklist based on the CARE (CAse REport) guidelines (Gagnier JJ et al., 2013; www.care-statement.org). "Reported" status: Yes = fully addressed; Partial = addressed but incomplete/placeholder pending; No = not included; NA = not applicable.

Item	Topic / Checklist item	Reported	Location in manuscript & notes
**Title, key words & abstract**			
**1**	Title — the words "case report" appear in the title along with the phenomenon of greatest interest.	**Yes**	Title. Includes "A Case Report"; phenomenon = lower-moiety UPJO in an incomplete duplex system presenting as Dietl's crisis, treated with robot-assisted pyeloplasty.
**2**	Key words — 2–5 key words identifying diagnoses/interventions.	**Yes**	Keywords. Seven terms (Dietl's crisis; lower-moiety UPJO; incomplete duplex; bifid ureter; crossing vessels; robot-assisted pyeloplasty; MAG3).
**3a**	Abstract — Introduction: what is unique and why it is important.	**Yes**	Abstract, Background/Objectives.
**3b**	Abstract — main concerns and important clinical findings.	**Yes**	Abstract, Case Presentation.
**3c**	Abstract — primary diagnoses, interventions, and outcomes.	**Yes**	Abstract, Case Presentation.
**3d**	Abstract — conclusion / main take-away lessons.	**Yes**	Abstract, Conclusions.
**Introduction**			
**4**	Introduction — brief background with references to relevant literature.	**Yes**	Section 1 (Introduction), references [1–23].
**Patient information & clinical findings**			
**5a**	Patient information — de-identified patient-specific information.	**Yes**	Section 2.1. 14-year-old boy; no identifiers included.
**5b**	Patient information — primary concerns and symptoms.	**Yes**	Section 2.1 (recurrent right flank colic triggered by heavy fluid intake, nausea, vomiting).
**5c**	Patient information — medical, family, psychosocial history (incl. genetic).	**Partial**	Section 2.1. No prenatal hydronephrosis, medications, allergies, prior surgery, or relevant family history. Psychosocial/genetic history not addressed (not contributory).
**5d**	Patient information — relevant past interventions and outcomes.	**Yes**	Section 2.1. No prior urological surgery/intervention (none applicable).
**6**	Clinical findings — significant physical examination and clinical findings.	**Yes**	Sections 2.2 (examination; febrile UTI with leukocytosis, pyuria/bacteriuria, E. coli) and 2.3–2.4 (imaging/functional findings).
**Timeline**			
**7**	Timeline — episode of care organized as a timeline.	**Yes**	Table 1 (timeline of clinical course, work-up, and management).
**Diagnostic assessment**			
**8a**	Diagnostic assessment — diagnostic methods.	**Yes**	Sections 2.2–2.4: ultrasonography, CT urography, 99mTc-MAG3 diuretic renography, laboratory testing and urine culture.
**8b**	Diagnostic assessment — diagnostic challenges.	**Yes**	Sections 2.2–2.4 and Discussion: normal interval ultrasound, intermittent obstruction, moiety localization within duplex anatomy.
**8c**	Diagnostic assessment — diagnostic reasoning and other diagnoses considered.	**Yes**	Discussion (differential diagnosis paragraph): nephrolithiasis, pyelonephritis, nonobstructive hydronephrosis, musculoskeletal and GI causes.
**8d**	Diagnostic assessment — prognostic characteristics (e.g., staging) where applicable.	**Partial**	Sections 2.3–2.4: SFU grade 3 hydronephrosis (ultrasound); differential and moiety split renal function (ROI-dependent estimates); preserved overall function.

**Therapeutic intervention**			
**9a**	Therapeutic intervention — types of intervention.	**Yes**	Section 2.2 (antibiotics for febrile UTI) and 2.5 (robot-assisted dismembered Anderson–Hynes pyeloplasty; double-J stent).
**9b**	Therapeutic intervention — administration (dosage, strength, duration).	**Partial**	Sections 2.2/2.5: IV cefuroxime + gentamicin, 10-day course; 4-0 Vicryl anastomosis; 6 Fr × 26 cm double-J for 4 weeks. Antibiotic doses and MAG3 protocol fields (hydration/furosemide timing/bladder management) remain as placeholders to be completed.
**9c**	Therapeutic intervention — changes in intervention (with rationale).	**Yes**	Section 2.2: IV-to-oral cefuroxime de-escalation after culture results and clinical improvement.
**Follow-up and outcomes**			
**10a**	Follow-up and outcomes — clinician- and patient-assessed outcomes.	**Yes**	Section 2.6: resolution of symptoms, no recurrence of Dietl-type episodes at ~3 months.
**10b**	Follow-up and outcomes — important follow-up test results.	**Yes**	Section 2.6: ultrasound resolution; MAG3 no stasis; CTU normal drainage at ~3 months.
**10c**	Follow-up and outcomes — intervention adherence and tolerability.	**Partial**	Sections 2.5–2.6: uneventful recovery; stent removed at 4 weeks. Formal adherence/tolerability not separately assessed.
**10d**	Follow-up and outcomes — adverse and unanticipated events.	**Yes**	Sections 2.5–2.6: no intraoperative complications; uneventful postoperative course.
**Discussion**			
**11a**	Discussion — strengths and limitations.	**Yes**	Section 3, including the dedicated Limitations paragraph.
**11b**	Discussion — relevant medical literature with references.	**Yes**	Section 3 (references [1–23]).
**11c**	Discussion — scientific rationale for conclusions (incl. possible causes).	**Yes**	Section 3: crossing vessels as a likely contributor to intermittency; anatomy-driven selection of dismembered repair.
**11d**	Discussion — primary take-away lessons (one-paragraph conclusion, no references).	**Yes**	Section 4 (Conclusions).
**Patient perspective & informed consent**			
**12**	Patient perspective — the patient shares their perspective whenever possible.	**No**	Not included. Consider adding a brief patient/parent perspective statement if obtainable, or note that it was not provided.
**13**	Informed consent — patient/guardian gave informed consent.	**Yes**	Backmatter, Informed Consent Statement: written consent obtained from the parent/guardian; provided to the journal if requested.

Items requiring author attention before submission: (i) Item 12 — add a patient/parent perspective if available; (ii) Items 9b/8 — complete the MAG3 protocol fields (hydration method, furosemide timing, bladder management, acquisition duration) and antibiotic dosing currently left as placeholders in the manuscript.

Reference: Gagnier JJ, Kienle G, Altman DG, Moher D, Sox H, Riley D; CARE Group. The CARE guidelines: consensus-based clinical case reporting guideline development. 2013.
